# Impact of HCV viremia on HBV biomarkers in patients coinfected with HBV and HCV

**DOI:** 10.1186/s12879-022-07326-1

**Published:** 2022-04-09

**Authors:** Chih-Wei Tseng, Wen-Chun Liu, Chi-Yi Chen, Ting-Tsung Chang, Kuo-Chih Tseng

**Affiliations:** 1 Department of Internal Medicine, Dalin Tzu Chi Hospital, Buddhist Tzu Chi Medical Foundation, No 2, Ming-Shen Road, Dalin Town, Chia-Yi County, 622 Taiwan; 2 School of Medicine, Tzuchi University, Hualien, Taiwan; 3 Department of Internal Medicine, National Cheng Kung University Hospital, Tainan, Taiwan; 4 College of Medicine, National Cheng Kung University, Tainan, Taiwan; 5 Center of Infectious Disease and Signaling Research, National Cheng Kung University, Tainan, Taiwan; 6 Department of Internal Medicine, Ditmanson Medical Foundation Chia-Yi Christian Hospital, Chia-Yi, Taiwan

**Keywords:** Hepatitis B surface antigen, Hepatitis B core-related antigen, HBV pregenomic RNA, HBV/HCV coinfection

## Abstract

**Backgrounds:**

Hepatitis B virus (HBV) biomarkers reflect the status of HBV infection; however, their role in patients with chronic hepatitis B and C (HBV/HCV) coinfection remains unknown. This study evaluated the characteristics of HBV biomarkers in patients with chronic HBV/HCV coinfection.

**Methods:**

One hundred untreated HBV/HCV coinfected patients were enrolled. Active viral infection was defined as viral load above 2000 U/L and 15 U/L for HBV and HCV, respectively. Blood samples were analyzed for HBV biomarkers, including hepatitis B surface antigen (HBsAg), hepatitis B core-related antigen (HBcrAg), HBV DNA, and HBV pregenomic RNA (HBV pgRNA). The impact of HCV viremia was also studied.

**Results:**

A total of 15 patients were HBV-inactive/HCV-inactive, 63 patients were HBV-inactive/HCV-active, 14 patients were HBV-active/HCV-inactive and 8 patients were HBV-active/HCV-active. A total of 71 (71%) patients were active HCV and 22 (22%) were active HBV. HBsAg, HBcrAg, and HBV DNA correlated with each other (*P* < 0.001). HBV pgRNA displayed no correlations with HBV DNA, HBsAg, or HBcrAg. Patients with HCV viremia had significantly lower HBV DNA, HBsAg, and HBcrAg levels as well as higher HBV pgRNA levels and lower HBV DNA:pgRNA ratio than those without viremia (HBV DNA, *P* < 0.001; HBsAg, *P* = 0.015; HBcrAg, *P* = 0.006; HBV pgRNA, *P* = 0.073; and HBV DNA:pgRNA ratio, *P* < 0.001).

**Conclusions:**

In patients coinfected with HBV and HCV, HBsAg, HBcrAg, and HBV DNA significantly correlated with each other. HBV and HCV coinfected patients with HCV viremia have lower HBV DNA, HBsAg, HBcrAg, and HBV DNA:pgRNA ratio as well as higher HBV pgRNA levels.

## Background

The prevalence of hepatitis B virus (HBV) and hepatitis C virus (HCV) coinfection is not uncommon in high epidemic areas because they share common routes of transmission [1, 2]. Patients with HBV/HCV coinfection have a higher risk of advanced liver disease, cirrhosis, and hepatocellular carcinoma (HCC) than those with monoinfection [1, 3]. This population also carries the risk of HBV DNA reactivation following HCV direct-acting antiviral treatment [4]. Hence, understanding the viral interactions and molecular aspects of HBV/HCV coinfection are important for treatment.

Patients with HBV/HCV coinfection show a large spectrum of virological profiles, which demonstrates the complexity of the interaction between the two viruses [5, 6]. The most common clinical scenario among coinfected patients is HCV predominance with high HCV RNA levels and low HBV DNA levels; some patients experience HBV predominance with high HBV DNA levels and low HCV RNA levels, while others present alternating phases of dominance of one virus over the other [3].

Since the presence of HCV viremia may suppress HBV DNA, the status of HBV infection cannot be evaluated with HBV DNA simply due to the persistence of covalently closed circular DNA (cccDNA) in the nuclei of infected hepatocytes [5, 6]. Although liver biopsy is the most accurate technique for quantification of intrahepatic cccDNA, its utility is limited by its invasive nature, the potential for sampling error, and the lack of a standardized assay [7, 8]. Several non-invasive biomarkers, including hepatitis B surface antigen (HBsAg), hepatitis B core-related antigen (HBcrAg), and HBV pregenomic RNA (HBV pgRNA), have been developed to reflect the activity of intrahepatic cccDNA [7, 9]. Growing evidence supports the serum HBV DNA correlated with serum HBsAg, HBcrAg, and HBV pgRNA in patients without antiviral treatment [9–11]. These biomarkers also serve as surrogate markers to reflect the cccDNA activity in virally suppressed patients with low detectable HBV DNA under nucleos(t)ide analogues (NA) therapy [12–15]. However, the above evidence does not disclose the characteristics of HBV biomarkers in HBV/HCV coinfected patients.

The aim of this study was to investigate the presentation of and correlations among HBV biomarkers in patients with HBV/HCV coinfection. The impact of HCV viremia on HBV biomarkers was also evaluated.

## Methods

### Subjects

This study was conducted between May 2017 to July 2019 at Dalin Tzu Chi Hospital, Buddhist Tzu Chi Medical Foundation, Chiayi, Taiwan. We included the patients (> 20 years old) who were seropositive for HBsAg and the anti-HCV antibody (anti-HCV) for more than 6 months at the time of enrollment into the study. The exclusion criteria included patients who were < 20 years old, with antiviral treatment history, human immunodeficiency virus infection, hepatitis delta virus infection, sclerosing cholangitis, autoimmune hepatitis, primary biliary cirrhosis, α1-antitrypsin deficiency, Wilson’s disease, overt hepatic failure, or HCC. Finally, total 100 patients with chronic HBV/HCV coinfection were enrolled.

Accordioning to current guidelines, the cutoff vale using to define the HBeAg(–) patients with active CHB and those who required treatment was 2000 IU/mL [16, 17]. Therefore, we used above 2000 IU/mL as the definition of active HBV infection in this study. The HCV patients with detectable viremia required anti-HCV treatment [18, 19]. In this study, we used the COBAS^®^ AmpliPrep/COBAS^®^ TaqMan^®^ HCV Test v2.0 (Roche Diagnostics, Rotkreuz, Switzerland) with a lower limit of quantification of 15 IU/mL. Active HCV infection was defined as a virus load above 15 IU/mL [1]. According to these definitions, the cohort was divided into four different groups: HBV-inactive/HCV-inactive group (BICI), HBV-inactive/HCV-active group (BICA), HBV-active/HCV-inactive group (BACI), and HBV-active/HCV-active group (BACA).

The study was approved by the Ethics Committee of Dalin Tzu Chi Hospital (approval number B10901004). All patients signed informed consent forms before commencement of the study.

### Clinical monitoring

All subjects received regular follow-up at the outpatient department. Blood samples were collected at regular clinic visits for evaluating levels of HBsAg, hepatitis B e antigen (HBeAg), HBcrAg, HBV pgRNA, HBV DNA, anti-HCV, HCV RNA, and HCV genotype. Serum aspartate aminotransferase (AST), alanine aminotransferase (ALT), total bilirubin, albumin, estimated glomerular filtration rate (eGFR), prothrombin time, platelets, and alpha-fetoprotein (AFP) were measured by using commercially available assays. The abdominal ultrasonography was done at the time of enrollment into the study. Fibrosis-4 (FIB-4) was used for non-invasive liver fibrosis assessment [20]. The cirrhosis of liver was determined by radiologic cirrhosis or FIB-4 more than 3.25 [21]. Radiologic cirrhosis was defined as coarse liver echotexture with nodularity and small liver size or the features of portal hypertension (e.g., ascites, splenomegaly, varices and collateral circulation of hepatic portal system) based on imaging findings [22]. A diagnosis of fatty liver was based on results from abdominal ultrasound, including higher liver echogenicity compared to right kidney, deep attenuation, and vessel blurring [23].

### HBV/HCV quantification and HCV genotyping

HBV DNA was quantified using the COBAS^®^ HBV quantitative nucleic acid test on the COBAS^®^ 4800 System (Roche Diagnostics, Rotkreuz, Switzerland), with a lower limit of quantification of 5 IU/mL. HCV RNA levels were determined by using the COBAS^®^ AmpliPrep/COBAS^®^ TaqMan^®^ HCV Test v2.0 (Roche Diagnostics) with a lower limit of quantification of 15 IU/mL. HCV genotyping was performed using the COBAS^®^ HCV GT assay (Roche Diagnostics).

### HBsAg and HBcrAg quantification

HBsAg and HBcrAg was quantified using the a fully automated chemiluminescent enzyme immunoassay system (CLEIA) (Lumipulse G1200; Fujirebio, Inc., Tokyo, Japan) in a Lumipulse G1200 automated analyzer (Fujirebio, Inc., Tokyo, Japan). The linear detection range of the HBsAg-HQ ranged from 5 to 150,000 mIU/mL. This HBcrAg assay had a lower limit of detection of 2.0 log U/mL and a linear range of 3.0–7.0 log U/mL (1 kU/mL is equal to 3 log U/mL).

### Extraction and reverse transcription of HBV pgRNA

HBV RNA was extracted from 150 µL of serum using the Total RNA Extraction Miniprep System Kit according to the manufacturer’s instructions (Viogene, Taipei, Taiwan) and treated with DNase I (Thermo Fisher Scientific, Waltham, MA, USA). Isolated HBV RNA was reverse transcribed to complementary DNA using RevertAid reverse transcriptase (Thermo Fisher Scientific, Waltham, MA, USA) with an HBV specific RT primer for HBV pgRNA [24]. The complementary DNA samples were held at 4 °C before proceeding to quantitative real-time polymerase chain reaction.

### Quantification of serum HBV pgRNA

The levels of serum HBV RNA were detected by qPCR in LightCycler 480 II Real-time PCR Detection System (Roche, Mannheim, Germany) with a SYBR Green method [24]. The limit of detection of serum HBV pgRNA was 1466 copies/mL, as calculated by probit analysis [24]. For statistical analysis, those serum samples with HBV pgRNA below limit of detection or not detected were recorded as 1465 copies/mL (3.17 log copies/mL).

### Statistical analysis

Statistical analysis was performed using SPSS Statistics version 19.0 software (SPSS Inc., Chicago, IL, USA). Categorical variables were expressed as frequency count and percent of total. The Chi-square test or Fisher’s exact test was used for comparison of categorical data, as appropriate. Continuous variables were described as mean ± standard deviation. The Mann–Whitney *U* test was used to compare differences in continuous variables between groups. In addition, linear regression analysis was performed to evaluate the correlations between HBV biomarkers. A positive β coefficient indicated a positive association between HBV biomarkers. A *P* value < 0.05 was considered significant.

## Results

### Demographic and laboratory features of the study subjects

A total of 100 HBV/HCV coinfected patients were enrolled in this study. There were 37 men and 63 women, and the mean patient age was 62.3 years (SD: 10.7 years) (Table 1). A total of 15 patients were HBV-inactive/HCV-inactive, 63 patients were HBV-inactive/HCV-active, 14 patients were HBV-active/HCV-inactive and 8 patients were HBV-active/HCV-active. A total of 71 (71%) patients were active HCV and 22 (22%) were active HBV. All of the included patients were seronegative for HBeAg, except one patient with HBeAg-positive CHB in the BICA group.

**Table 1:** Clinical and virological characteristics of the patients

	Total (n = 100)	BICI (n = 15)	BICA (n = 63)	BACI (n = 14)	BACA (n = 8)	*P* value
Age (years)^†^	62.3 ± 10.7	58.8 ± 12.4	63.6 ± 10.7	57.9 ± 9.1	65.5 ± 6.4	0.144
Male (n, %)	37 (37.0%)	4 (26.7%)	24 (38.1%)	4 (28.6%)	5 (62.5%)	0.337
Cirrhosis (n, %)	27 (27.0%)	0 (0.0%)	21 (33.3%)	2 (14.3%)	4 (50.0%)	0.018
Fatty liver (n, %)	35 (35.0%)	5 (33.3%)	22 (34.9%)	7 (50.0%)	1 (12.5%)	0.364
Alcoholism	14 (14.0%)	1 (6.7%)	9 (14.3%)	1 (7.1%)	3 (37.5%)	0.180
HCV RNA (log IU/mL)^†^	4.5 ± 2.3	1.0 ± 0.0	5.9 ± 0.9	1.0 ± 0.0	5.8 ± 0.5	< 0.001
Genotype (n, %)						< 0.001
Type 1	45 (45.0%)	0 (0.0%)	38 (60.3%)	0 (0.0%)	7 (87.5%)	
Type 2	22 (22.0%)	0 (0.0%)	21 (33.3%)	0 (0.0%)	1 (12.5%)	
Type 6	4 (4.0%)	0 (0.0%)	4 (6.3%)	0 (0.0%)	0 (0.0%)	
HBV DNA (log IU/mL)^†^	2.3 ± 1.5	1.9 ± 0.8	1.5 ± 0.7	4.6 ± 0.9	4.8 ± 1.5	< 0.001
HBsAg (log IU/mL)^†^	1.0 ± 2.1	1.4 ± 1.9	0.5 ± 2.1	1.9 ± 1.8	2.3 ± 0.7	0.004
HBcrAg (log IU/mL)^†^	3.2 ± 0.6	3.2 ± 0.5	3.1 ± 0.5	3.6 ± 0.7	3.5 ± 1.2	0.008
HBV pgRNA (copies/mL)^†^	4.1 ± 1.2	3.7 ± 1.2	4.2 ± 1.2	3.9 ± 1.1	4.9 ± 1.6	0.119
FIB-4^†^	2.5 ± 2.1	1.4 ± 0.5	2.7 ± 2.4	2.1 ± 1.8	3.5 ± 1.7	0.008
Total bilirubin (mg/dL)^†^	0.7 ± 0.3	0.7 ± 0.3	0.7 ± 0.4	0.9 ± 0.3	0.7 ± 0.3	0.166
ALT (U/L)^†^	65.2 ± 55.0	35.7 ± 25.2	73.6 ± 55.2	29.8 ± 9.5	106.4 ± 79.5	< 0.001
AST (U/L)^†^	48.4 ± 40.5	28.3 ± 18.1	52.6 ± 36.6	26.9 ± 9.3	91.2 ± 80.2	< 0.001
Albumin (g/dL)^†^	4.3 ± 0.3	4.5 ± 0.2	4.3 ± 0.4	4.3 ± 0.2	4.2 ± 0.2	0.123
Prothrombin time (s)^†^	10.8 ± 0.7	10.7 ± 0.3	10.7 ± 0.8	11.1 ± 0.6	10.7 ± 0.7	0.172
AFP (U/L)^†^	28.3 ± 192.8	3.2 ± 2.4	40.7 ± 241.4	3.9 ± 4.4	16.0 ± 24.7	0.035
Platelets (× 10^3^/mm^3^)^†^	186.2 ± 58.2	205.5 ± 45.6	183.0 ± 59.99	192.4 ± 58.4	163.9 ± 62.1	0.432
eGFR^†^	86.6 ± 24.7	82.5 ± 16.3	85.0 ± 25.5	100.7 ± 26.7	86.6 ± 24.7	0.194

BICI, HBV-inactive/HCV-inactive group; BICA, HBV-inactive/HCV-active group; BACI, HBV-active/HCV-inactive group; BACA, HBV-active/HCV-active group (BACA); HCV, hepatitis C virus; HBV, hepatitis B virus; HBsAg, hepatitis B surface antigen; HBcrAg, hepatitis B core-related antigen; pgRNA, pregenomic RNA; FIB-4, fibrosis-4 index; ALT, alanine aminotransferase; AST, aspartate aminotransferase; AFP, alpha-fetoprotein; eGFR, estimated glomerular filtration rate

^†^Data are expressed as mean ± standard deviation

Based on viral load, patients were placed into one of the following groups: BICI (n = 15; 15.0%), BICA (n = 63; 63.0%), BACI (n = 14; 14.0%), or BACA (n = 8; 8.0%). The demographic, clinical, and virological characteristics of each group are presented in Table 1. HBV DNA, HBsAg, HBcrAg, and HCV RNA showed statistically significant differences among the four groups while HBV pgRNA did not. Liver enzyme (ALT/AST), cirrhosis, FIB-4, and AFP levels in the BICA and BACA groups were higher than those in the BICI and BACI groups. There were no statistically significant differences among the four groups regarding age, sex, fatty liver, alcoholism, albumin, total bilirubin, eGFR, prothrombin time, and platelet count.

### HBV biomarkers of the patients with HBV/HCV coinfection

**Fig. 1:**
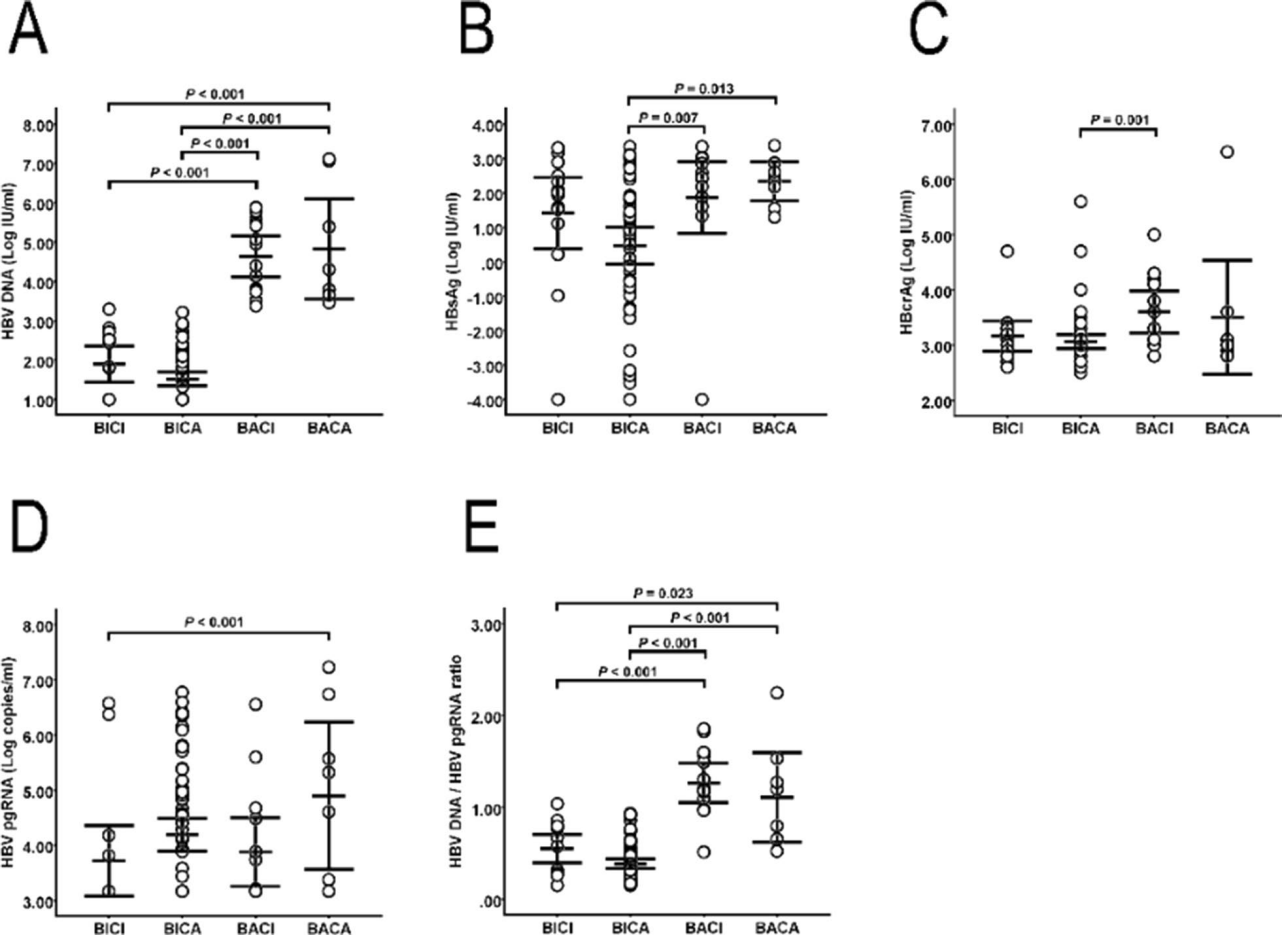
Expression of HBV DNA, HBsAg, HBcrAg, and HBV pgRNA in in patients coinfected with HBV/HCV (n = 100). **A** HBV DNA was significantly higher in the HBV-active groups (**A**; BACA vs. BICA, *P* < 0.001; BACA vs. BICI, *P* < 0.001; BACI vs. BICA, *P* < 0.001; and BACI vs. BICI, *P* < 0.001). **B** Serum HBsAg in the HBV-active groups was significantly higher than that in the BICA group (**B**; BACA vs. BICA, *P* = 0.013; and BACI vs. BICA, *P* = 0.007). **C** Serum HBcrAg in the BACI group was higher than that in BICA group (**C**; *P* = 0.001). **D** HBV pgRNA in the BACA group was significantly higher than that in the BICI group (**D**; *P* < 0.001). **E** The HBV DNA:HBV pgRNA ratio was significantly higher in the BACA and BACI groups than in the BICI and BICA groups (**E**; BACA vs. BICA, *P* < 0.001; BACA vs. BICI, *P* = 0.023; BACI vs. BICA, *P* < 0.001; and BACI vs. BICI, *P* < 0.001). HBV, hepatitis B virus; HCV, hepatitis C virus; HBsAg, hepatitis B surface antigen; HBcrAg, hepatitis B core-related antigen; pgRNA, pregenomic RNA

Figure 1 shows the HBV biomarkers (including HBV DNA, HBsAg, HBcrAg, and HBV pgRNA) of each of the four groups. The HBV-active groups (BACA and BACI) had significantly higher level of HBV DNA (Fig. 1A; BACA vs. BICA, *P* < 0.001; BACA vs. BICI, *P* < 0.001; BACI vs. BICA, *P* < 0.001; BACI vs. BICI, *P* < 0.001). Levels of serum HBsAg in the HBV-active groups (BACA and BACI) were significantly higher than that in the BICA group (Fig. 1B; BACA vs. BICA, P = 0.013; BACI vs. BICA, P = 0.007). The serum HBcrAg level in the BACI group was higher than that in the BICA group (Fig. 1C; *P* = 0.001). In addition, the level of HBV pgRNA in the BACA group was significantly higher than that in the BICI group (Fig. 1D; *P* = 0.001); however, there was no significant difference in the HBV pgRNA level among the four groups (Table 1; *P* = 0.119). Interestingly, using HBV DNA divided by HBV pgRNA, the HBV DNA:HBV pgRNA ratio was significantly higher in BACA and BACI than in BICI and BICA (Fig. 1E; BACA vs. BICA, *P* < 0.001; BACA vs. BICI, *P* = 0.023; BACI vs. BICA, *P* < 0.001; BACI vs. BICI, *P* < 0.001).

### Correlations between HBV biomarkers of the patients with HBV/HCV coinfection

**Fig. 2:**
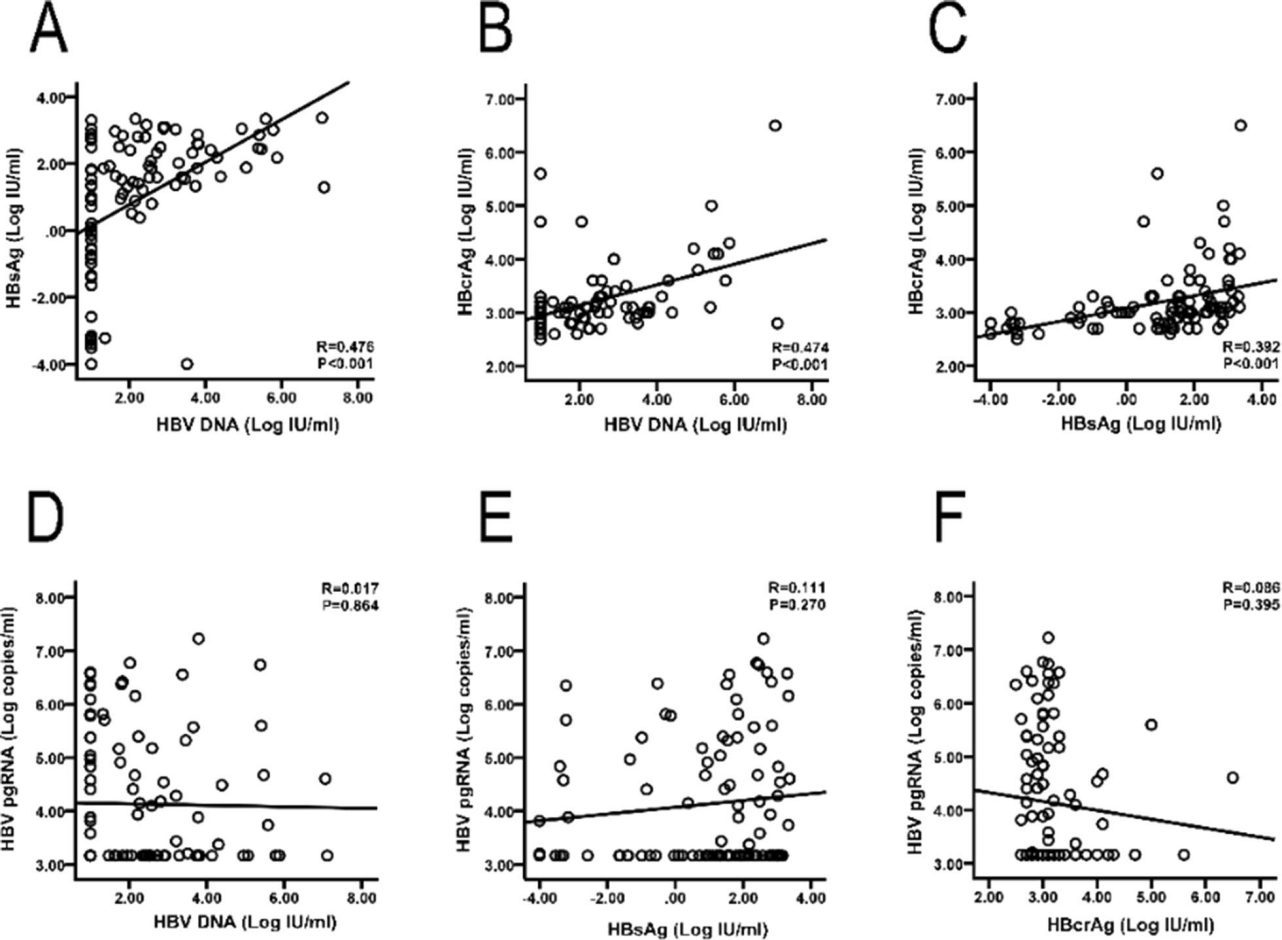
Linear regression analyses for the correlations between HBV biomarkers in patients coinfected with HBV/HCV. **A** HBV DNA was significantly correlated with HBsAg (**A**; R = 0.476, R^2^ = 0.227, β = 0.463, and *P* < 0.001). **B** HBV DNA was significantly correlated with HBcrAg (**B**; R = 0.474, R^2^ = 0.225, β = 0.474, and *P* < 0.001). **C** HBsAg also showed a positive correlation with HBcrAg (**C**; R = 0.392, R^2^ = 0.153, β = 0.392, and *P* < 0.001). **D**–**F** HBV pgRNA had no correlations with HBV DNA, HBsAg, or HBcrAg (**D**–**F**; HBV DNA, R = 0.017, R^2^ = 0.0003, β = − 0.017, and *P* = 0.864; HBsAg, R = 0.111, R^2^ = 0.012, β = 0.111, and *P* = 0.270; and HBcrAg, R = 0.086, R^2^ = 0.0037, β = − 0.086, and *P* = 0.395, respectively). HBV, hepatitis B virus; HCV, hepatitis C virus; HBsAg, hepatitis B surface antigen; HBcrAg, hepatitis B core-related antigen; pgRNA, pregenomic RNA

Linear regression analyses demonstrated positive linear correlations between HBsAg, HBcrAg, and HBV DNA. HBV DNA significantly correlated with HBsAg (Fig. 2A; R = 0.476, R^2^ = 0.227, β = 0.463, and *P* < 0.001) and HBcrAg (Fig. 2B; R = 0.474, R^2^ = 0.225, β = 0.474, and *P* < 0.001). HBsAg also showed a positive correlation with HBcrAg (Fig. 2C; R = 0.392, R^2^ = 0.153, β = 0.392, and *P* < 0.001). However, HBV pgRNA showed no correlations with HBV DNA, HBsAg, and HBcrAg (Fig. 2D–F; HBV DNA, R = 0.017, R^2^ = 0.0003, β = − 0.017, and *P* = 0.864; HBsAg, R = 0.111, R^2^ = 0.012, β = 0.111, and *P* = 0.270; and HBcrAg, R = 0.086, R^2^ = 0.0037, β = − 0.086, and *P* = 0.395, respectively).

### HBV biomarkers in HBV/HCV coinfected patients with and without HCV viremia

**Fig. 3:**
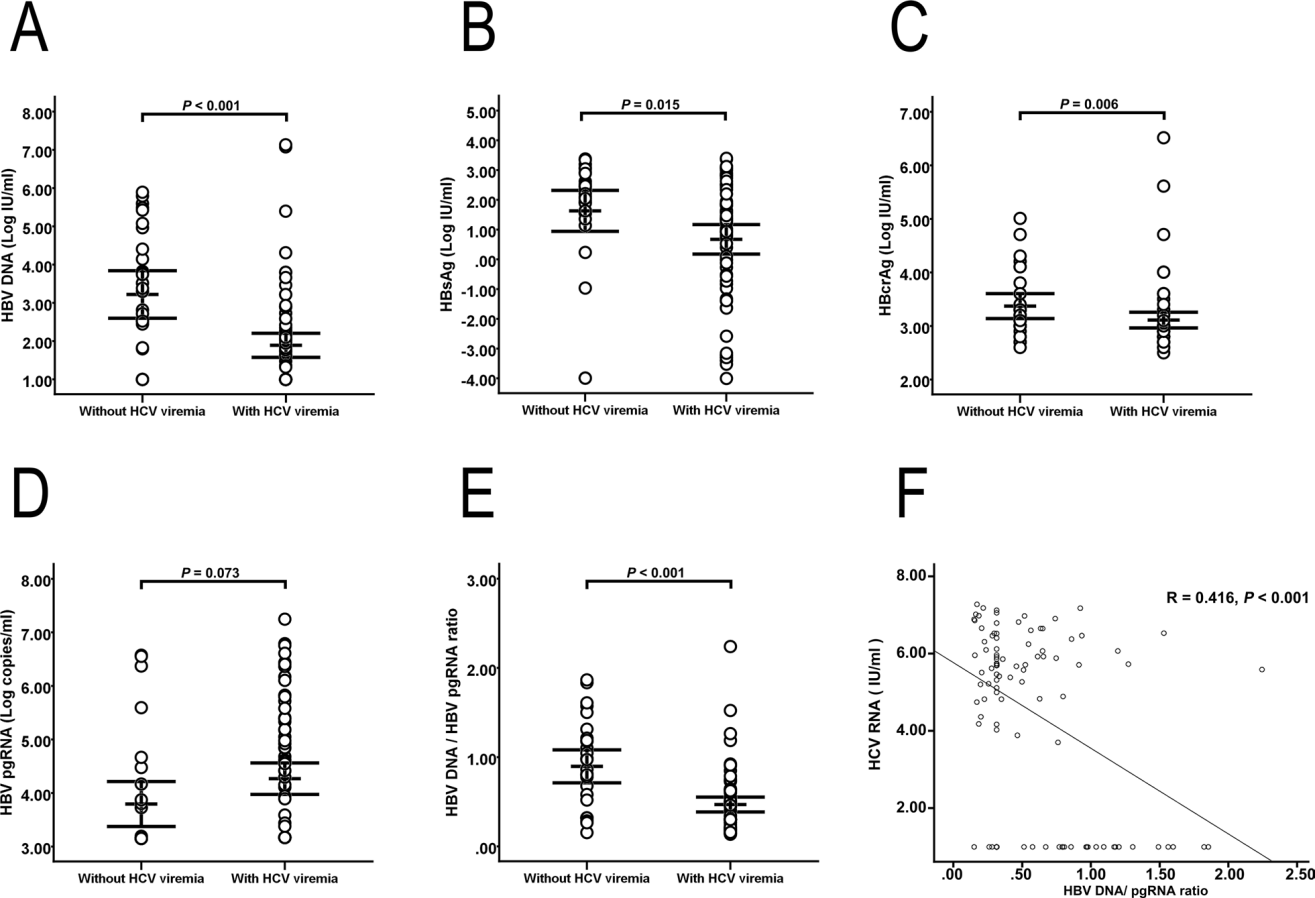
HBV biomarker expression levels in chronic HBV/HCV coinfected patients with (n = 71) and without (n = 29) HCV viremia. **A**–**C** The HBV DNA, HBsAg and HBcrAg levels of patients with HCV viremia were significantly lower than those of patients without viremia (**A**–**C**; HBV DNA, *P* < 0.001; HBsAg, *P* = 0.015; and HBcrAg, *P* = 0.006, respectively). **D** The HBV pgRNA level of patients with viremia trended higher than that of patients without viremia (**D**; *P* = 0.073). **E** Patients with viremia had a lower HBV DNA:HBV pgRNA ratio (**E**; *P* < 0.001). **F** The amount of HCV RNA had a negative correlation between HCV RNA and HBV DNA/pgRNA ratio (Fig. 2A; R = 0.416, R^2^ = 0.173, β = – 0.416, and *P* < 0.001). HBV, hepatitis B virus; HCV, hepatitis C virus; HBsAg, hepatitis B surface antigen; HBcrAg, hepatitis B core-related antigen; pgRNA, pregenomic RNA

In HBV/HCV coinfected patients with HCV viremia, HBV DNA, HBsAg, and HBcrAg were significantly reduced than in those without viremia (Fig. 3A–C; HBV DNA, *P* < 0.001; HBsAg, *P* = 0.015; and HBcrAg, *P* = 0.006, respectively). Although the difference was not statistically significant, patients with viremia exhibited higher HBV pgRNA levels than those without viremia (Fig. 3D; *P* = 0.073). Interestingly, patients with viremia had a lower HBV DNA:HBV pgRNA ratio than those without viremia, indicating lower reverse transcription efficiency of pgRNA in patients with viremia (Fig. 3E; *P* < 0.001). The amount of HCV RNA had a negative correlation between HCV RNA and HBV DNA/pgRNA ratio (Fig. 3F; R = 0.416, R^2^ = 0.173, β = – 0.416, and *P* < 0.001).

## Discussion

The present study demonstrated that HBV DNA, HBsAg, and HBcrAg show linear positive correlations with each other in HBV/HCV coinfected patients. However, HBV pgRNA levels were not associated with HBV DNA, HBsAg, or HBcrAg. The presence of HCV viremia appeared to suppress HBV DNA, HBsAg, HBcrAg, and indicated an increasing level of HBV pgRNA. The lower HBV DNA:HBV pgRNA ratio in patients with HCV viremia indicated ineffective reverse transcription of HBV pgRNA.

In line with previous epidemiology reports, the current study determined that active HCV infection plays a dominant role in patients with HBV/HCV coinfection (Table 1) [2, 25]. Active HCV status in patients with HBV/ HCV coinfection correlated with higher extent of liver injury (ALT/AST), higher fibrosis scores (FIB-4), and enhanced cirrhosis (Table 1). However, the association between high HBV DNA (active HBV) and patient characteristics (such as liver injury or cirrhosis) was not significant. This observation suggests that identification of non-invasive biomarkers, other than HBV DNA, is necessary to reflect the transcriptional activity of intrahepatic viruses and immune response.

Several reports have demonstrated that serum HBV DNA correlates with serum HBsAg, HBcrAg, and HBV pgRNA during the natural course of hepatitis B viral infection [26–29]. One recent meta-analysis including 5591 patients without antiviral therapy found a close correlation between HBcrAg and HBV DNA [10]. The optimal cut-offs (log U/mL) were 3.6 and 4.5 to diagnose HBV DNA levels (IU/mL) of ≥ 2000 and ≥ 20,000, respectively [10]. The levels of serum HBV pgRNA strongly correlate with serum HBV DNA in treatment-naïve patients, but the correlation becomes weaker upon receiving NAs [30]. As for presentation in patients with HBV monoinfection, our study showed that HBsAg and HBcrAg levels changed in parallel with the HBV DNA level in the HBV/HCV coinfected patients (Fig. 2). Positive linear correlations between HBsAg, HBcrAg, and HBV DNA were identified (Fig. 2). In contrast, lack of an association between serum HBV pgRNA and other HBV biomarkers was noted in our study (Fig. 2D–F). Theoretically, both serum HBV DNA and HBV pgRNA can be useful markers for assessing HBV cccDNA activity in treatment-naïve patients. However, HBV DNA is suppressed due to halted HBV pgRNA reverse transcription by HCV viremia [31, 32], making HBV pgRNA a more direct marker for cccDNA. Thus, serum HBV pgRNA may be a surrogate marker for intrahepatic HBV cccDNA in patients with HBV/HCV coinfection compared to serum HBV DNA, HBsAg, and HBcrAg. However, further studies are warranted to clarify this observation.

The persistence of serum HBV pgRNA levels raises interest in the role of HCV viremia in HBV/HCV coinfected patients. In general, HCV is the dominant virus that actively replicates and suppresses the replication of HBV [5, 33]. The mechanism of this interaction is not well-understood, although several hypotheses regarding direct or indirect (mediated by various host immune responses) viral interference have been proposed to explain the dominant role of HCV. A number of studies have suggested that HBV suppression is mediated by the HCV core protein [31, 32]. In coinfection, the HCV core protein was found to form a complex with HBV polymerase and impede its function [31, 32]. Another hypothesis states that HCV infection activates interferon production within the hepatocytes, thereby suppressing HBV further due to its antiviral effects [3, 5]. Our study revealed that HCV viremia is associated with low HBV DNA, HBsAg, and HBcrAg. These results suggest that HCV may suppress HBV polymerase and impair mRNA translation, which reduces the production of HBV DNA, HBsAg, and HBcrAg in patients with HBV/HCV coinfection.

In contrast with the suppression of HBV DNA, HBsAg, and HBcrAg, our study also discovered a trend of higher HBV pgRNA associated with HCV viremia (including in the BACA and BICA groups) (Fig. 3D; *P* = 0.073). In HBV replication cycle, circular DNA genome of mature HBV from the linear pgRNA template takes place inside the core [34]. After HBV polymerase protein binding to the epsilon (ε) loop at the 5′ end of pgRNA, assembly of HBV begins with packaging of pgRNA into immature nucleocapsids (NC), which are converted to mature NCs containing the genomic relaxed circular (RC) DNA as a result of reverse transcription [35]. However, a number of studies have investigated that HCV core protein could complex with HBV polymerase and impede its function [5, 31, 36]. Therefore, HBV pgRNA virion levels increased in BACA and BICA groups might result from blocking the reverse transcription activity of HBV DNA polymerase while HCV core protein capture HBV polymerase. In general, the levels of serum HBV pgRNA strongly correlate with serum HBV DNA in treatment-naïve patients [30]. The lower level of HBV pgRNA is reasonable in the BICI and BICA groups due to the low HBV replication activity [37]. However, HCV viremia increased the level of HBV pgRNA in the BICA group and caused the BICA group to have comparable HBV pgRNA levels to the BACA and BACI groups (Fig. 1D). Combined with the effects of HBV viremia and HCV viremia, the BACA group with both viremia effects had higher HBV pgRNA levels than the BICI group with both negative viremia effects (Fig. 1D; *P* < 0.001). Further studies are necessary to address the correlation between HCV viremia and HBV pgRNA.

Combined serum HBV DNA and HBV pgRNA levels are better indicators of cccDNA activity and the HBV DNA:HBV pgRNA ratio reflects the HBV polymerase protein reverse transcription activity [38]. The current study demonstrated that HCV viremia leads to a lower HBV DNA:HBV pgRNA ratio that is compatible with the suppression of HBV polymerase by HCV [31, 32]. Several studies have reported that NAs or pegylated interferon alpha therapy can effectively block reverse transcription and suppress HBV viral replication, causing an inversion of the HBV DNA:HBV RNA ratio [39, 40]. As the presentation of anti-HBV therapy, the current study demonstrated that patients with HCV viremia present a lower HBV DNA level and show a higher serum HBV pgRNA level than those without viremia. Lower HBV DNA:HBV pgRNA ratio further indicated lower reverse transcription efficiency of pgRNA in patients with HCV viremia.

There were several limitations in this study. First, the case number of some groups was small due to the nature of virological profiles. Most patients were HCV-dominant. However, this study remains the largest cohort study on this subject. This report adds new information about the characteristics of HBV biomarkers in patients with HBV/HCV coinfection. Second, our results do not provide information regarding the molecular mechanism of the increase in HBV pgRNA levels in patients with HCV viremia. Further studies are needed to clarify these aspects. Third, we compared the dynamic change of most HBV biomarkers, including HBsAg, HBV DNA, HBcrAg and HBV pgRNA in the current study. However, the analysis of other circulating HBV biomarkers such as microRNA or HBV nucleic acid-related antigen were not done. Further study maybe required.

Despite these limitations, this study has several strengths. Although there are many studies about the HBV biomarkers in HBV patients, this is the first study with the comprehensive investigation of various HBV biomarkers in HBV/HCV coinfected patients. Second, it is interesting to note that HBV pgRNA levels increase, especially when HCV proliferation is active. This is not mentioned in previous reports. Third, the relatively high HBV pgRNA level, low HBV DNA and low HBV DNA:HBV pgRNA ratio suggested that HCV viremia may be responsible for the decrease of HBV pgRNA RT activity. The results suggested that those HBV biomarkers have the potential as the predictive biomarkers of HBV reactivation following HCV direct-acting antiviral treatment in patients with HBV/HCV coinfection.

## Conclusions

The current study revealed that HBV DNA, HBsAg, and HBcrAg significantly correlated with each other in patients coinfected with HBV and HCV. In addition, HBV and HCV coinfected patients with HCV viremia have lower HBV DNA, HBsAg, HBcrAg, and HBV DNA:pgRNA ratio as well as higher HBV pgRNA than those without viremia. The possible involvement of HCV RNA in HBV mRNA translation and pgRNA reverse transcription activity needs further investigation.

## Data Availability

The datasets used in the current study are available from the corresponding author on reasonable request.

## Abbreviations

HBV: Hepatitis B virus
HCV: Hepatitis C virus
HCC: Hepatocellular carcinoma
cccDNA: Covalently closed circular DNA
HBsAg: Hepatitis B surface antigen
HBcrAg: Hepatitis B core-related antigen
HBV pgRNA: HBV pregenomic RNA
NA: Nucleos(t)ide analogues
anti-HCV: Anti-hepatitis C antibody
HBeAg: Hepatitis B e antigen
AST: Aspartate aminotransferase
ALT: Alanine aminotransferase
eGFR: Estimated glomerular filtration rate
AFP: Alpha-fetoprotein
FIB-4: Fibrosis-4
NC: Nucleocapsids
